# T8. NEUROLOGICAL SOFT SIGNS (NSS) IN SCHIZOPHRENIA: AN UPDATE ON THE STATE- VERSUS TRAIT-PERSPECTIVE

**DOI:** 10.1093/schbul/sby016.284

**Published:** 2018-04-01

**Authors:** Silke Bachmann, Johannes Schröder

**Affiliations:** 1 Clienia Littenheid; 2 University of Heidelberg

## Abstract

**Background:**

Neurological soft signs (NSS) represent minor neurological signs which indicate non-specific cerebral dysfunction. Numerous studies have confirmed their presence in schizophrenia across all stages of the disease. NSS have been looked at as an endophenotype or a trait phenomenon for many decades. However, during the past years studies increasingly reported on fluctuations of the NSS scores. To shade further light on the question whether NSS represent a state or a trait component or both, a review of longitudinal studies on schizophrenia patients was performed, because only measurements at two or more points in time can answer the question at hand.

**Methods:**

A search of studies which had assessed NSS in adult schizophrenia patients and included at least one follow-up examination was undertaken. Studies which had been published between January 1966 and June 2017 and listed in relevant databases were included. Due to the fact, that ongoing brain maturation lasts until adulthood and is paralleled by a loss of those NSS which are present in childhood, studies on teenagers were excluded.

**Results:**

Twenty-nine follow-up studies were identified which overall used well-known instruments for the investigation of NSS. Patients were at different disease stages. All expressed abnormally increased NSS, however to different extents. An NSS reduction during the course was detected in most first episode patients and those with a remitting course whereas chronically ill patients exhibited stable or increasing NSS scores. The change over time could for the most part be attributed to changes in the motor system subscales and to a lesser amount to sensory integration scales. As opposed to the earlier notion that medication evokes or worsens NSS, studies largely agreed on a positive interrelation between medication response and improvement of NSS. The type of antipsychotic was of small importance and when side-effects were commented on there was a weak relationship with NSS. On the other hand, studies gave some hints at relationships between NSS and symptoms, i.e. negative and cognitive symptoms.

**Discussion:**

The reviewed studies confirmed the presence of abnormal, i.e. elevated NSS in patients diagnosed with schizophrenia. Studies disagreed on the amount of abnormalities in NSS. However, subgroups emerged with either stable or fluctuating properties of NSS. The latter speaks very much in favor of a state-trait dichotomy being present in NSS and thus challenges the view that NSS depict an endophenotype.

